# How do policy tool combinations drive the construction of public health technology R&D alliances?

**DOI:** 10.3389/fpubh.2026.1759788

**Published:** 2026-02-06

**Authors:** Yangchun Cao, Jing Zhang, Ling Ning

**Affiliations:** School of Management, Guangdong Ocean University, Zhanjiang, China

**Keywords:** evolutionary game, evolutionary path, policy tools, public health, technology R&D alliance

## Abstract

To effectively respond to public health emergencies, establishing an efficient technology R&D alliance is critically important. This study develops a tripartite evolutionary game model involving the government, pharmaceutical enterprises, and academic and research institutions to examine how a combination of supply-side, demand-side, and environmental-side policy tools drives the formation of such alliances. The findings reveal that demand-side government procurement exerts the strongest incentive effect on enterprise and institutional participation, outperforming supply-side and environmental-side measures. Furthermore, policy intensity exhibits a scientifically discernible threshold: excessive intervention may not only increase fiscal pressure on the government but also paradoxically reduce willingness to participate due to diminishing marginal returns. Consequently, optimizing the mix of policy tools and implementing differentiated, targeted incentives are essential for fostering high-efficiency public health technology R&D alliances. This study offers a dynamic analytical framework and evidence-based guidance for policymakers in designing effective collaborative innovation strategies.

## 1 Introduction

Innovating the management of public health technology R&D is a key measure to strengthen the national emergency response system and, more fundamentally, a critical foundation for building a robust national health system (1). During the fight against the COVID-19 pandemic, China leveraged an integrated public health technology R&D alliance and fully capitalized on institutional advantages to achieve globally recognized outcomes. In the face of complex and rapidly evolving health threats, establishing sustained, strategically coordinated R&D alliances has become imperative; enhancing their operational efficiency, collaborative capacity, and governance of scientific resources now constitutes a core priority (2). In major public health emergencies, it is crucial to explore the rapid response mechanism of R&D alliances to achieve a ‘race against time'. This requires the swift identification and accurate understanding of unknown causes behind public health emergencies, as well as the initiation of R&D projects within a short period. It also involves accelerating the R&D process while adhering to scientific principles and ensuring safety, followed by proposing reasonable and effective countermeasures (3). For instance, conventional vaccine development typically takes 8 to 10 years from concept to clinical use (4). However, in response to the COVID-19 pandemic in 2020, R&D organizations in multiple countries compressed this cycle to just 12 months. Unfortunately, many other public health emergencies that have occurred in recent years—such as major outbreaks caused by the Zika virus and Ebola virus—did not see efficient R&D alliances promoting timely vaccine or drug development. Therefore, establishing high-quality public health R&D alliances promptly remains a persistent global challenge (5, 6).

The public health technology R&D alliance shares characteristics of general R&D alliances, such as a focus on innovation in biotechnology, life sciences, and other related fields, but also exhibits traits like long R&D cycles, high uncertainty, and a strong demand for new knowledge (7). In problem-oriented, application-driven contexts, these alliances are often expected to generate actionable results within days or months, with contingency plans sometimes needed within hours (8). This necessitates a departure from conventional research pathways while maintaining scientific rigor under extreme time constraints and high costs of trial and error (9). Therefore, optimizing multi-party collaboration is essential, that is, tactically harnessing the innovative strengths and initiatives of individual participants (10), while strategically leveraging national institutional capacities and policy tools to integrate research capabilities effectively (11). Maximizing the catalytic role of policy tools is crucial for responding to public health crises and fostering resilient technological R&D alliances.

Building on this foundation, this study seeks to address a central research question: how can the government integrate and leverage multidimensional policy tools to effectively incentivize pharmaceutical enterprises and academic and research institutions to actively participate, thereby promoting the formation of robust public health technology R&D alliances? By developing a tripartite evolutionary game model involving the government, enterprises, and academic and research institutions, this paper systematically examines the dynamic effects of supply-side, demand-side, and environmental-side policy tools on the strategic decisions of each stakeholder and the evolution of system equilibrium. The primary contributions of this study are threefold. First, it systematically applies the classic three-category framework of policy tools—supply, demand, and environment—to the context of collaborative innovation during public health emergencies, thereby refining the theoretical analytical framework in this domain. Second, through the evolutionary game model, it reveals the behavioral adjustment mechanisms of alliance participants under the influence of multiple policy dimensions, offering insights into the dynamics of cooperation and coordination. Third, numerical simulations demonstrate that demand-side policies, particularly government procurement, exert a significantly stronger incentive effect, and further indicate that each type of policy tool exhibits an optimal intervention range. These findings provide actionable guidance for policymakers in designing precise and effective incentive packages.

## 2 Literature review

Policy tools serve as concrete mechanisms for achieving policy objectives, and their selection and combination directly influence governance effectiveness. The classic tripartite framework introduced by Rothwell and Zegveld (12)—comprising supply-side, demand-side, and environmental-side tools—offers a structured and analytically valuable approach to policy analysis. Rather than simply cataloging policy tools, this framework highlights how different tools exert systemic effects by intervening at distinct stages of the innovation process. Subsequent research has emphasized that individual tools often have limited impact; instead, the strategic combination of tools and their synergistic interactions are critical to policy success (13, 14). These interactions may manifest as complementarity or synergy, but they can also lead to conflict or crowding-out effects, underscoring the importance of assessing the combined impacts of policy mixes (15). While this framework has been extensively applied in industrial and environmental policy domains (16), its systematic application in the context of collaborative innovation during public health emergencies remains underdeveloped. Although existing studies on public health crisis responses recognize the significance of policy support (17), they often fail to systematically analyze interventions through the lens of the tripartite framework. As a result, the relative effectiveness and interaction dynamics of various policy tools in facilitating rapid R&D alliances remain poorly understood. This theoretical gap contributes to policy design that is frequently based on experiential judgment rather than rigorous, evidence-based scholarship.

As a distinctive form of cooperative organization designed to address extreme uncertainty and time pressure, public health technology R&D alliances encounter unique governance challenges. Existing research has acknowledged their necessity (18), and scholarly consensus holds that the success of such alliances hinges on the effective integration of resources and capabilities across heterogeneous actors—including governments, enterprises, and academic and research institutions (19). Although contemporary studies have moved beyond static analyses of alliance structures, such as organizational models, participant configurations, institutional arrangements, or ex-post performance assessments, and have increasingly focused on how these alliances dynamically evolve from loosely connected networks to tightly coordinated systems under exogenous policy shocks (20), systematic and in-depth inquiry remains limited. Specifically, while we understand the characteristics of high-performing alliances and recognize the importance of policy support, there is still a dearth of robust theoretical frameworks that elucidate the mechanisms through which policy tools influence the strategic decisions of key stakeholders and ultimately catalyze alliance formation. This absence of a process-oriented analytical lens constrains our capacity to predict and optimize the timing and modes of policy intervention. Therefore, this gap constitutes a critical entry point for the present study. The evolutionary game model developed in this paper seeks to bridge this void by mapping the full causal chain: policy tool combination; micro-level strategic interactions among actors; macro-level alliance evolution.

Evolutionary game theory relaxes the stringent assumption of “perfect rationality” found in classical game theory, offering a robust analytical framework for examining the long-term dynamic processes through which boundedly rational agents adapt their strategies via mechanisms such as imitation, trial and error, and learning (21). This approach has been widely applied across various domains of public governance, including studies on vaccination strategies, public health responses to natural disasters, and the sustainable use of public resources (22–24). These applications demonstrate that evolutionary game models effectively capture the evolutionary trajectories and stable equilibria of collective behavior under policy interventions. Nevertheless, opportunities remain to advance this body of research by applying evolutionary games to a specific yet underexplored context: public health technology R&D alliances. On the one hand, although tri-party or even multi-party game models exist in the literature (23, 25), few studies focus explicitly on the core “government–pharmaceutical enterprise–academic and research institution” triadic structure or provide a thorough analysis of its inherent goal conflicts and synergy dynamics during public health emergencies. On the other hand, many existing models treat policy intervention at a relatively macro level, failing to systematically disaggregate policy tools along key dimensions—such as supply, demand, and environment—into quantifiable and comparable parameters within the modeling framework. Therefore, there is a need for an extended evolutionary game model that not only centers on the strategic interactions among government, enterprises, and academic and research institutions but also explicitly incorporates the combined effects of multidimensional policy tools, thereby enhancing our understanding of how differentiated policy levers influence alliance formation processes.

## 3 Construction of a game model for the public health technology R&D alliance

### 3.1 The operational mechanism of a game subject driven by policy tools

**Figure 1:**
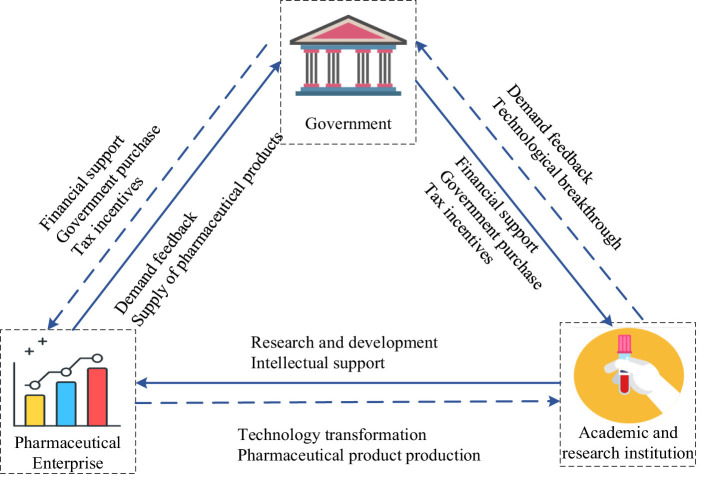
Operation mechanism of public health technology R&D alliance driven by policy tools.

The public health technology R&D alliance is an innovative platform focused on public health events, driven by the imperative to safeguard people's lives and wellbeing. It is led by the government, with participation from pharmaceutical enterprises, and coordination from academic and research institutes (26). The government guides the establishment and operation of the public health technology R&D alliance through policy tools. By organizing interdisciplinary teams for scientific and technological cooperation and coordinating science and technology with clinical prevention and control efforts, as well as closely aligning the science and technology governance system with government, industry, academia, research, and application sectors, this aims to promote project research and development, technology transformation, and application of achievements. Therefore, the public health technology R&D alliance embodies typical characteristics of being government-driven. The interaction mechanism among public health subjects can be deconstructed from three dimensions: subject structure, policy mechanism, and factor input and effect (as shown in Figure 1).

In terms of the subject structure, the main body of the public health technology R&D alliance includes the government, pharmaceutical enterprises, and academic and research institutions. This reflects an innovative relationship where the government leads, pharmaceutical enterprises participate, and academic and research institutions cooperate. In response to major public health emergencies, the state quickly mobilizes superior scientific and technological resources from various sectors such as pharmaceutical enterprises and academic and research institutions. This forms a public health technology R&D alliance that swiftly invests in emergency research projects. The organization of the technology R&D alliance reflects typical characteristics of the government's use of a policy mix to drive collaboration among multiple public health stakeholders (27).

In terms of policy mechanisms, the government builds and guides the operation and evolution of public health technology R&D alliances through a multi-level and systematic combination of policy tools. According to the policy classification model of Rothwell and Zegved (12), policy tools in the field of public health can be divided into three categories: supply side, demand side, and environmental side. They work in synergy to form the core mechanism that drives alliance construction, resource integration, and efficiency output. Supply-side policy tools focus on financial support, technological support, and talent development. The government directly addresses key element gaps in the R&D cycle of the alliance by setting up special research funds, building public experimental platforms, sharing data resources, and organizing cross-disciplinary research teams, thereby reducing innovation risks and accelerating technological breakthroughs. Demand-side policy tools, centered on government procurement, provide clear market access and technology transfer pathways for alliance members through centralized procurement of medical supplies, testing equipment, and scientific and technological research services, directly encouraging enterprises and academic research institutions to engage in emergency R&D and production. Meanwhile, as a key user, the government guides the R&D direction to closely align with actual demands by establishing technical standards and performance requirements. Environmental-side policy tools focus on institutional incentives and ecological development. By implementing preferential policies such as additional deductions for R&D expenses, tax reductions and exemptions, and expedited approval processes for pharmaceutical enterprises and academic institutions, a policy environment conducive to long-term collaboration and continuous innovation is created (28). These three types of policy tools, driven by the government, jointly shape the evolution of the alliance from emergency response to long-term development, promoting the continuous optimization of the science and technology governance system featuring the deep integration of “government, industry, academia, research, and application”.

In terms of factor input, government policy support is the key resource input for the public health technology R&D alliance. The government has played a leading role in the rapid response mechanism for emergency science and technology research, including capital investment in infrastructure construction, coordinated deployment of project personnel, and platform development for information resource collection. Academic and research institutions provide intellectual support such as talent, technology, patents, and essential experimental instruments and equipment for drug vaccine research and development. They also offer technical solutions for sudden public health events. Pharmaceutical enterprises leverage the supply capacity of the industrial chain to quickly bring drug vaccine products to market based on technological R&D results from academic and research institutions. Hospitals and related clinical rescue institutions use these drug vaccines to promptly treat patients during public health emergencies (29). In terms of revenue effect, rapid response and timely processing of public health emergencies can ensure regional social stability while improving social welfare and governance performance. By participating in the public health technology R&D alliance, academic and research institutions can enhance the quality of personnel training as well as the efficiency of scientific and technological achievement transformation. Pharmaceutical enterprises are able to convert scientific and technological innovation into products while enhancing overall value through improvements in the industrial chain (30).

### 3.2 Fundamental assumption

Based on the analysis of the operational mechanism of the public health technology R&D alliance, it is evident that the successful operation of the public health technology R&D alliance requires active participation from various stakeholders, including the government, pharmaceutical enterprises, academic and research institutions, and other relevant entities. Each stakeholder has its own intrinsic motivations and resistance toward promoting the establishment of a technology R&D alliance due to their resource dependence on one another. Motivations are primarily driven by anticipated benefits, while resistance stems from expected costs. The decision to promote the construction of a public health technology R&D alliance should be based on a comprehensive consideration of both benefits and costs. Therefore, this paper focuses on analyzing the government, pharmaceutical enterprises, and academic and research institutions to construct an evolutionary game model for the public health technology R&D alliance. Based on this analysis, we propose the following hypothesis.

**Hypothesis 1:** The public health technology R&D alliance involves participation from the government, pharmaceutical enterprises, and academic and research institutions. The core interest of the game subjects in this alliance is the limited rationality of these entities. Throughout their involvement in the public health technology R&D alliance, the government, pharmaceutical enterprises, and academic and research institutions dynamically adjust their strategic choices through mutual learning due to information acquisition constraints and resource limitations. This allows them to maximize their own utility while participating in the alliance.
**Hypothesis 2:** Within the technology R&D alliance, the government, pharmaceutical enterprises, and academic and research institutions will dynamically adjust their strategies in response to changes in the actual situation until system equilibrium is achieved. Each participant has two selection strategies: the strategy set for government departments is (positive incentive, negative incentive); the strategy set for pharmaceutical enterprises is (active participation, negative participation); and the strategy set for academic and research institutions is (active participation, negative participation). The initial income of the government, pharmaceutical enterprises, and academic and research institutions is denoted as g, e, and s, respectively. The probability that the three parties choose to actively participate in the technology R&D alliance is represented by *x, y, z*, where *x, y, z* ε [0, 1].
**Hypothesis 3:** Government. The public health technology R&D system has undergone a historical change from initial government “unified” management to social “diversified” coordination. With the participation of multiple stakeholders in the public health technology R&D alliance, the role of government departments has shifted from leader to guide (31). When the government utilizes policy tools to provide positive incentives, on the supply side, direct investment in funds is made for enterprises and research institutions that have joined emergency science and technology research; on the demand side, the government purchases developed pharmaceutical products; on the environmental side, tax incentives are provided to enterprises and research institutions actively participating in emergency science and technology research based on their income. It is assumed that under positive incentives, the total cost of manpower and material resources invested by the government is cb_1_ (the sum of supply, demand, and environmental policy costs), while enhancing credibility through health prevention and control results in benefits W_1_ for the government. In contrast, if the government does not actively implement incentives for public health emergency science and technology research, only market mechanisms will sustain the public health technology R&D alliance. Without policy tool incentives, this approach will lead to a loss of credibility for B_1_, threating the prevention and control risks faced by governments.
**Hypothesis 4:** Pharmaceutical enterprises play a crucial role in the public health technology R&D alliance, participating in public health emergency technology services through market-oriented means. The products provided by pharmaceutical enterprises include drug research and production, medical equipment and industry platform supply, and the construction of public health service facilities, from which they derive economic benefits for their operation and development. Assuming that pharmaceutical enterprises actively participate in the public health technology R&D alliance, the production costs incurred for communication, equipment, personnel, etc., are represented as cb_2_, while the economic benefits obtained through market transformation are denoted as W_2_. On the supply side, pharmaceutical enterprises receive financial support from the government, denoted as k_1_; on the demand side, the government procures market-based products for an amount of *p*. The corresponding income received by pharmaceutical enterprises is λ*p* (where λ represents the market profit ratio of pharmaceutical enterprises in government procurement). From an environmental perspective, based on the sales volume of pharmaceutical enterprise products, tax incentives are provided by the government. Assuming that *r* is the government's tax incentive coefficient, the amount of tax incentive enjoyed by pharmaceutical enterprises is *rW*_2_. On the other hand, when pharmaceutical enterprises passively participate in the public health technology R&D alliance, they do not generate additional income and incur losses *B*_2_ due to reputational damage and opportunity costs.
**Hypothesis 5:** Academic and research institutions leverage their technological R&D advantages to actively participate in the technological breakthroughs of public health technology, playing a crucial role in the research and development of drugs and vaccines. It is assumed that when actively participating in the public health technology R&D alliance, the R&D cost of personnel and equipment invested is represented as *cb*_3_, with the economic benefits obtained through market transformation denoted as *W*_3_. On the supply side, academic and research institutions also receive financial support from the government, with a funding scale of *k*_2_. On the demand side, academic and research institutions share policy benefits of government procurement with pharmaceutical enterprises; thus, the corresponding income obtained by academic and research institutions is (1 – λ)*p*. From an environmental perspective, based on the economic benefits generated by academic and research institutions, the government provides tax incentives; consequently, the amount of tax incentive enjoyed by academic and research institutions is *rW*_3_. Conversely, when academic and research institutions are not actively involved in the public health technology R&D alliance, they do not generate additional benefits but incur losses represented as *B*_3_ due to reputational damage and opportunity costs.

### 3.3 Model construction

According to the hypothesis, it is concluded that the evolutionary game income matrix of the three parties under the strategy set of (positive incentive, negative incentive), (active participation, negative participation), and (active participation, negative participation) is shown in Table 1. Analysis of the evolutionary stability strategy of public health subjects.

**Table 1:** Game payoff matrix.

**Strategy selection**			**Academic and research institution**	
**Government**	**Pharmaceutical enterprise**		**Active participation (** ***z*** **)**	**Passive participation (1 –** ***z*****)**
	Positive motivation (*x*)	Active participation (*y*)	*g*+*W*_1_−*k*_1_−*k*_2_−*p*−*rW*_2_−*rW*_3_ *e*+*W*_2_+*k*_1_+λ*p*+*rW*_2_−*c*_*b*2_; *s*+*W*_3_+*k*_2_+(1−λ)*p*+*rW*_3_−*c*_*b*3_	*g*+*W*_1_−*k*_1_−*p*−*rW*_2_; *e*+*W*_2_+*k*_1_+*p*+*rW*_2_−*c*_*b*2_; *s*−*B*_3_
		Passive participation (1 – *y*)	*g*+*W*_1_−*k*_2_−*p*−*rW*_3_; *e*−*B*_2_; *s*+*W*_3_+*k*_2_+*p*+*rW*_3_−*c*_*b*3_	*g*+*W*_1_; *e*−*B*_2_; *s*−*B*_3_
	Negative motivation (1 – *x*)	Active participation (*y*)	*g*−*B*_1_; *e*+*W*_2_−*c*_*b*2_; *s*+*W*_3_−*c*_*b*3_	*g*−*B*_1_; *e*+*W*_2_−*c*_*b*2_; *s*−*B*_3_
		Passive participation (1 – *y*)	*g*−*B*_1_; *e*−*B*_2_; *s*+*W*_3_−*c*_*b*3_	*g*−*B*_1_; *e*−*B*_2_; *s*−*B*_3_

## 4 Analysis of evolutionary stability strategy of public health subject

Based on the evolutionary game matrix, the expected revenue and average expected revenue of the three parties—government, pharmaceutical enterprises, and academic and research institutions—are calculated, and then the replication dynamic equation for each subject is constructed.

### 4.1 Analysis of government evolutionary stability strategy

According to the evolutionary game matrix and evolutionary game tree regarding whether the government actively promotes the formation of technology R&D alliances, the expected returns corresponding to different government strategic choices are calculated. Subsequently, the replication dynamic equation of the government's evolutionary game is constructed. The expected return and average expected return are as follows: *U*_11_, *U*_12_ obtained by the government's active promotion or negative and non-active promotion of the technology R&D alliance formation strategy, and the average expected returns $\bar{U_{1}}$.


$$\begin{array}{l} U_{11}=yz(g+W_{1}-k_{1}-k_{2}-p-rW_{2}-rW_{3})+y(1-z)(g\\ \;+\;W_{1}-k_{1}-p-rW_{2})+(1-y)z(g+W_{1}-k_{2}-p-rW_{3})\\ \;+(1-y)(1-z)(g+W_{1}) \end{array}$$ (1)

$$\begin{array}{l} U_{12}=yz(g-B_{1})+y(1-z)(g-B_{1})+(1-y)z(g-B_{1})\\ \;+(1-y)(1-z)(g-B_{1}) \end{array}$$ (2)

$$\begin{array}{lll} \bar{U_{1}} & = & xU_{11}+\left(1-x\right)U_{12} \end{array}$$ (3)

According to evolutionary game theory, the replication dynamic equation and the first-order partial derivative of the government's promotion of technology R&D alliance behavior can be expressed as follows:


$$\begin{array}{l} F(x)=\frac{dx}{dt}=x(U_{11}-U_{1})=x(x-1)(k_{1}y-W_{1}-B_{1}+zk_{2}\\ \;+py+pz+ryW_{2}+ryW_{3}-yzp) \end{array}$$ (4)

$$\begin{array}{l} F^{\prime }(x)=(2x-1)(k_{1}y-W_{1}-B_{1}+zk_{2}+py+pz+ryW_{2}\\ \;+ryW_{3}-yzp \end{array}$$ (5)

Suppose μ(*y*) = *k*_1_*y*−*W*_1_−*B*_1_+*zk*_2_+*py*+*pz*+*ryW*_2_+*ryW*_3_−*yzp*. According to the stability theorem of differential equations, the probability that the government actively promotes emergency science and technology research should be satisfied in a stable state: *F*(*x*) = 0 and *F*′(*x*) <0 due to$\frac{\partial \mu \left(y\right)}{\partial y}>0$. That is, μ(*y*) is an increasing function with respect to *y*. Therefore, when$y=y^{*}=\frac{W_{1}+B_{1}-zk_{2}-pz-\lambda zW_{3}}{k_{1}+p+\lambda W_{2}-zP}$, μ(*y*) = 0. The government's stability strategy cannot be determined. When *y*<y^*^, μ(*y*) <0, so ${\frac{dF\left(x\right)}{dx}|}_{x=1}<0$. At this time, *x* = 1 is the stable strategy point. To further analyze the important factors that affect the government's incentive strategy, the first-order partial derivatives of each variable y^*^ are obtained, respectively, $\frac{\partial y^{*}}{\partial W_{1}}>0$,$\frac{\partial y^{*}}{\partial B_{1}}>0$, $\frac{\partial y^{*}}{\partial W_{3}}<0$,$\frac{\partial y^{*}}{\partial k_{2}}<0$, $\frac{\partial y^{*}}{\partial p}<0$, $\frac{\partial y^{*}}{\partial k_{1}}<0$, $\frac{\partial y^{*}}{\partial W_{2}}<0$. Therefore, the smaller the values of *k*_1_, *k*_2_, *p, W*_2_, *W*_3_, or the larger the values of *B*_1_, *W*_1_, the greater the y^*^, making it easier to meet the conditions. The government tends to actively use policies to promote public health entities to conduct scientific and technological research. Based on the above analysis, the following inference can be made.

**Corollary 1:** The likelihood of the government promoting active participation in public health technology R&D alliances is inversely related to the government's input costs and procurement from pharmaceutical enterprises and academic and research institutions for economic gains through collaborative R&D. It is also negatively associated with the loss of credibility in adverse situations, the opportunity cost of prevention and control, and positively linked to enhancing government credibility.

### 4.2 Analysis of enterprise evolutionary stable strategy

According to the evolutionary game matrix and evolutionary game tree regarding whether enterprises actively participate in public health technology R&D alliances, the expected returns corresponding to different strategic choices of enterprises are calculated, and then the replication dynamic equation of the enterprise evolutionary game is constructed. Therefore, the expected returns*U*_21_ and *U*_22_ obtained by enterprises actively or passively participating in the technology R&D alliance, and the average expected returns $\bar{U_{2}}$ are


(6) $$\begin{array}{l} U_{21}=xz(\begin{array}{c} e+W_{2}+k_{1}+\lambda p+rW_{2}-c_{b2} \end{array})+x(1-z)(e+W_{2}\\ \;+k_{1}+p+rW_{2}-c_{b2})+(1-x)z(\begin{array}{c} e+W_{2}-c_{b2} \end{array})\\ \;+(1-x)(1-z)(e+W_{2}-c_{b2}) \end{array}$$

(7) $$\begin{array}{l} U_{22}=xz(\begin{array}{c} e-B_{2} \end{array})+x(1-z)\begin{array}{c} \left(\begin{array}{c} e-B_{2} \end{array})+(1-x)z(\begin{array}{c} \begin{array}{c} e-B_{2} \end{array} \end{array}\right) \end{array}\\ \;+(1-x)(1-z)(\begin{array}{c} e-B_{2} \end{array}) \end{array}$$

$$\begin{array}{lll} \bar{U_{2}} & = & yU_{21}+\left(1-y\right)U_{22} \end{array}$$ (8)

According to evolutionary game theory, the replication dynamic equation and the first derivative of the behavior of enterprises promoting the public health technology R&D alliance are


(9) $$\begin{array}{l} F(y)=\frac{dy}{dt}=y(y-1)(c_{b2}-B_{2}-W_{2}-xk_{1}-px-rW_{2}x\\ \;+xzp-\lambda pxz) \end{array}$$

(10) $$\begin{array}{l} F^{\prime }(y)=(2y-1)(c_{b2}-B_{2}-W_{2}-xk_{1}-px-rW_{2}x+xzp\\ \;-\lambda pxz) \end{array}$$

Suppose φ(*z*) = *c*_*b*2_−*B*_2_−*W*_2_−*xk*_1_−*px*−*rW*_2_*x*+*xzp*−λ*pxz*. According to the stability theorem of differential equation, the probability of pharmaceutical enterprises actively participating in collaborative emergency science and technology research must be satisfied in a stable state. *F*(*y*) = 0 and *F*′(*y*) <0. Since $\frac{\varphi \left(z\right)}{\partial z}>0$, that is, φ(*z*)is an increasing function with respect to z. When $z=z^{*}=\frac{B_{2}-c_{b2}+W_{2}+xk_{1}+px+rW_{2}x}{px\left(1-\lambda \right)}$, φ(*z*) = 0. The stability strategy of pharmaceutical enterprises cannot be determined. When *z*<z^*^, ${\frac{dF\left(y\right)}{dy}|}_{y=0}<0$, *y* = 0 is the stable strategy point. To further explore the influencing factors that may affect the probability of pharmaceutical enterprises actively participating in public health technology R&D alliance, the first-order partial derivatives of each variable in z^*^ are calculated and can be obtained as $\frac{\partial z^{*}}{\partial c_{b2}}<0$,$\frac{\partial z^{*}}{\partial B_{2}}>0$,$\frac{\partial z^{*}}{\partial W_{2}}>0$,$\frac{\partial z^{*}}{\partial p}>0$,$\frac{\partial z^{*}}{\partial k_{1}}>0$, and $\frac{\partial z^{*}}{\partial \lambda }>0$. Therefore, when *B*_2_, *W*_2_, *k*_1_*, p*, and λ are larger, *c*__*b*_2_ is smaller, z^*^ is larger, the condition of *z*<z^*^ is easier to be established, and pharmaceutical enterprises are more inclined to actively participate in scientific and technological research alliance.

**Corollary 2:** The probability of pharmaceutical enterprises actively participating in public health technology R&D alliances is positively correlated with economic benefits, financial support funds, additional benefits provided by the government, and the proportion of reputation, opportunity cost loss, and market income under negative participation. It is negatively correlated with production costs such as communication, equipment, and personnel expenses incurred under active participation.

### 4.3 Analysis of enterprise evolutionary stable strategy

According to the evolutionary game matrix and evolutionary game tree regarding whether academic and research institutions are actively involved in public health technology R&D alliances, the expected benefits corresponding to different strategic choices of these institutions are calculated, and the replication dynamic equation of the evolutionary game for academic and research institutions is constructed.

The expected benefits obtained from the active or negative participation of research institutions in the public health technology R&D alliance are *U*_31_, *U*_32_, and the average expected returns $\bar{U_{3}}$:


(11) $$\begin{array}{l} U_{31}=xy(s+W_{3}+k_{2}+(1-\lambda )p+rW_{3}-c_{b3})+x(1-y)(s\\ \;+W_{3}+k_{2}+p+rW_{3}-c_{b3})+(1-x)y(s\\ \;+W_{3}-c_{b3})+(1-x)(1-y)(s+W_{3}-c_{b3}) \end{array}$$

(12) $$\begin{array}{l} U_{32}=xy(s-B_{3})+x(1-y)(s-B_{3})+(1-x)y(s-B_{3})+(1\\ \;-x)(1-y)(s-B_{3}) \end{array}$$

$$\begin{array}{lll} \bar{U_{3}} & = & zU_{31}+\left(1-z\right)U_{32} \end{array}$$ (13)

According to evolutionary game theory, the replication dynamic equation and the first-order partial derivative of the behavior of research institutions promoting the public health technology R&D alliance are formulated.


(14) $$\begin{array}{l} F(z)=\frac{dz}{dt}=z(U_{31}-\bar{U_{3}})=z(z-1)(c_{b3}-B_{3}-W_{3}-xk_{2}\\ \;-px-rxW_{3}+\lambda pxy) \end{array}$$

(15) $$\begin{array}{l} F^{\prime }(z)=(2z-1)(c_{b3}-B_{3}-W_{3}-xk_{2}-px-rxW_{3}\\ \;+\lambda pxy) \end{array}$$

Suppose 𝔐(*y*) = *c*_*b*3_−*B*_3_−*W*_3_−*xk*_2_−*px*−*rW*_3_*x*+λ*pxy*. According to the stability theorem of differential equation, the probability that the research institution chooses to actively participate in the public health technology R&D alliance must be satisfied in a stable state: *F*(*z*) = 0 and *F*′(*z*) <0. Since $\frac{\partial 𝔐\left(y\right)}{\partial y}>0$, 𝔐(*y*) is an increasing function with respect to *y*. At that time, $y=y^{**}=\frac{B_{3}-c_{b3}+W_{3}+xk_{2}+px+rxW_{3}}{\lambda px}$,𝔐(*y*) = 0 It is impossible to determine the stability strategy of academic and research institutions. When *y*>y^**^, 𝔐(*y*)>0, ${\frac{dF\left(z\right)}{dz}|}_{z=0}<0$, *z* = 0is the stable strategy point. When*y*<y^**^, 𝔐(*y*) <0, ${\frac{dF\left(z\right)}{dz}|}_{z=1}<0$, *z* = 1 is the stable strategy point. To further explore the influencing factors that may affect the probability of academic and research institutions actively participating in public health technology R&D alliance, the first-order partial derivatives of each variable are obtained $\frac{\partial y^{**}}{\partial B_{3}}>0$, $\frac{\partial y^{**}}{\partial c_{b3}}<0$, $\frac{\partial y^{**}}{\partial W_{3}}>0$, $\frac{\partial y^{**}}{\partial k_{2}}>0$, $\frac{\partial y^{**}}{\partial p}>0$, $\frac{\partial y^{**}}{\partial \lambda }<0$. Therefore, when *B*_3_, *W*_3_, *k*_2_, and *p* are larger, *c*__*b*_3_ and λ are smaller, y^*^ is smaller, the condition of *y*<y^**^ is easier to be established, and the research institutions are more inclined to actively participate in the public health technology R&D alliance.

**Corollary 3:** The probability of academic and research institutions actively participating in public health technology R&D alliances is positively correlated with economic benefits, financial support funds, additional benefits from government purchases, reputation, and opportunity cost losses from negative participation. It is negatively correlated with the production costs incurred during active participation and the market income ratio of pharmaceutical enterprises in government procurement.

### 4.4 Local stability analysis of the public health technology R&D system

Based on the above analysis, it is found that there is an interactive relationship among the probability of the government actively guiding and purchasing public health products *x*, the probability of pharmaceutical enterprises actively participating in the public health technology R&D alliance and providing technical support *y*, and the probability of academic and research institutions actively participating in the public health technology R&D alliance and providing intellectual support *z*. Let *F*(*x*) = 0, *F*(*y*) = 0, *F*(*z*) = 0 hold at the same time, eight local equilibrium points of system *D* can be obtained: H_1_(0, 0, 0), H_2_(1, 0, 0), H_3_(0, 1, 0), H_4_(0, 0, 1), H_5_(1, 1, 0), H_6_(1, 0, 1), H_7_(0, 1, 1), H_8_(1, 1, 1).

Referring to the evolutionary game analysis method proposed by Friedman, the local stability analysis of the Jacobian matrix of the replication dynamic system can further obtain the ESS of the system (32). According to the previous analysis, the dynamic equations for the government, pharmaceutical enterprises, and academic and research institutions can be obtained, along with the Jacobian matrix J of system D:


(16) $$J=\left[\begin{array}{ccc} \frac{\partial F(x)}{\partial x} & \frac{\partial F(x)}{\partial y} & \frac{\partial F(x)}{\partial z}\\ \frac{\partial F(y)}{\partial x} & \frac{\partial F(y)}{\partial y} & \frac{\partial F(y)}{\partial z}\\ \frac{\partial F(z)}{\partial x} & \frac{\partial F(z)}{\partial y} & \frac{\partial F(z)}{\partial z} \end{array}\right]=\left[\begin{array}{ccc} A_{11} & A_{12} & A_{13}\\ A_{21} & A_{22} & A_{23}\\ A_{31} & A_{32} & A_{33} \end{array}\right]$$

Of which:


(17) $$\{\begin{array}{l} A_{11}=(2x-1)(k_{1}y-W_{1}-B_{1}+k_{2}z+py+pz+rW_{2}y\\ +rW_{3}z-pyz)\\ A_{12}=x(x-1)(k_{1}+p+rW_{2}-pz)\\ A_{13}=x(x-1)(k_{2}+p+rW_{3}-py)\\ A_{21}=-y(y-1)(k_{1}+p+rW_{2}-pz+\lambda pz)\\ A_{22}=-(2y-1)(B_{2}-c_{b2}+W_{2}+k_{1}x+px+rW_{2}x\\ -pxz+\lambda pxz)\\ A_{23}=xy(p-\lambda p)(y-1)\\ A_{31}=-z(z-1)(k_{2}+p+rW_{3}-\lambda py)\\ A_{32}=\lambda pxz(z-1)\\ A_{33}=-(2z-1)(B_{3}-c_{b3}+W_{3}+K_{2}x+px+rW_{3}x\\ -\lambda pxy) \end{array}$$

According to the Lyapunov stability criterion and Reinhard's point of view (38), the equilibrium point is considered an evolutionary stable point of the system when all eigenvalues of the Jacobian matrix are negative real numbers, while the equilibrium point is unstable when at least one eigenvalue is a positive real number. Therefore, the eight local equilibrium points are substituted into the Jacobian matrix, and the eigenvalues corresponding to each equilibrium point can be obtained. The results are shown in Table 2.

**Table 2:** Stability analysis of equilibrium point.

**Point of equilibrium**	**Eigenvalues of Jacobian matrix**	
	λ_1_, λ_2_, λ_3_	**Real symbol**
H_1_(0, 0, 0)	*B*_1_+*W*_1_, *B*_2_−*c*_*b*2_+*W*_2_, *B*_3_−*c*_*b*3_+*W*_3_	(+, +, +)
H_2_ (1, 0, 0)	−*B*_1_−*W*_1_, *k*_1_−_*c*_*b*_2_+*B*_2_+*p*+*W*_2_+*rW*_2_, *k*_2_−*c*_*b*3_+*B*_3_+*P*+*W*_3_+*rW*_3_	(–, +, +)
H_3_ (0, 1, 0)	*B*_1_−*k*_1_−*p*+*W*_1_−*rW*_2_, *C*_2_−*L*_2_−*R*_2_, *L*_3_−*C*_3_+*R*_3_	(–, –,+)
H_4_ (0, 0, 1)	*B*_1_−*k*_2_−*p*+*W*_1_−*rW*_3_, *B*_2_−*c*_*b*2_+*W*_2_, *c*_*b*3_−*B*_3_−*W*_3_	(–, +, –)
H_5_ (1, 1, 0)	*k*_1_−*B*_1_+*P*−*W*_1_+*rW*_2_, *c*_*b*2_−*k*_1_−*B*_2_−*p*−*W*_2_−*rW*_2_, *k*_2_−*c*_*b*3_+*B*_3_+*p*+*W*_3_+*rW*_3_−λ*p*	(–,–, *N*)
H_6_ (1, 0, 1)	*k*_2_−*B*_1_+*p*−*W*_1_+*rW*_3_, *k*_1_−*c*_*b*2_+*B*_2_+*W*_2_+λ*p*+*rW*_2_, *c*_*b*3_−*k*_2_−*B*_3_−*p*−*rW*_3_	(–, +, –)
H_7_ (0, 1, 1)	*B*_1_−*k*_2_−*k*_1_−*p*+*W*_1_−*rW*_2_−*rW*_3_, *c*_*b*2_−*B*_2_−*W*_2_, *c*_*b*3_−*B*_3_−*W*_3_	(*N*, –, –)
H_8_ (1, 1, 1)	*k*_1_+*k*_2_−*B*_1_+*p*−*W*_1_+*rW*_2_+*rW*_3_, *c*_*b*2_−*k*_1_−*B*_2_−*W*_2_−λ*p*−*rW*_2_, *c*_*b*3_−*k*_2_−*B*_3_−*P*−*W*_3_−*rW*_3_+λ*p*	(*N*, –, –)

“+” indicates that the symbol is positive, “–” indicates that the symbol is negative, and “N” indicates that the symbol is uncertain.

According to the analysis of the eigenvalues of the Jacobian matrix in Table 2, the possible equilibrium state of the game system composed of the government, pharmaceutical enterprises, and academic and research institutions is analyzed in depth, as follows:

**Case 1:** When *k*_2_+*B*_3_+*p*+*W*_3_+*rW*_3_−λ*p*<*c*_*b*3_, the cost of active participation of research institutions in the alliance is greater than the benefits obtained, H_5_ (1,1,0) is the evolutionary stable point. At this time, the government actively uses policy tools, pharmaceutical enterprises actively participate, and academic and research institutions participate negatively.

**Case 2:** When *W*_1_+*B*_1_<*k*_2_+*k*_1_+*p*+*rW*_2_+*rW*_3_, the cost of the government's active promotion of public health technology R&D alliance exceeds the sum of the benefits in its positive state and the losses in its negative state, H_7_ (0, 1, 1) is the evolutionary stable point. At this point, the government passively promotes the public health technology R&D alliance, while pharmaceutical enterprises and academic and research institutions actively participate. Due to the complexity, variability, and diffusivity of sudden public health events, substantial funding and resource inputs are necessary. As the institution and implementer of public policy, the government must assume a leading role. In addition, while the government's policy tools can effectively encourage the active participation of pharmaceutical enterprises and academic and research institutions, it is crucial to remain vigilant against the risks of excessive government involvement, which can impose a financial burden that negatively impacts the continuity and stability of policy implementation.

**Case 3:** When *k*_1_+*k*_2_−*B*_1_+*p*+*rW*_2_+*rW*_3_<*W*_1_, the cost of the government's policy tools for actively promoting the public health technology R&D alliance is lower than its total revenue, H_8_ (1,1,1) is the stable point. At this time, the government actively uses policy tools to promote and guide stakeholders in carrying out technology R&D. Academic and research institutions actively engage in drug and vaccine research and development projects, while pharmaceutical enterprises focus on producing vaccines, drugs, and incubating scientific research initiatives. The active participation of multiple public health entities in technology R&D is the most effective measure for addressing sudden public health events and represents the ideal state. In this system, the “two-wheel drive” of the government and the market positively influences the active involvement of pharmaceutical enterprises and academic and research institutions in the construction of the public health technology R&D alliance, while the government also benefits from reasonable public return income.

## 5 Numerical analysis

Numerical simulation was carried out using MATLAB 2018a to comprehensively analyze the influence trajectory of factors such as policy tools on the evolutionary behavior of the game subjects from the supply-side, demand-side, and environment-side, respectively, and to obtain the stable strategy of the three-party game evolution of the public health technology R&D alliance. Considering the current situation and the difficulty in precisely quantifying the cost-to-benefit ratio of sudden public health events, as well as the challenges in obtaining objective data with a unified dimension, this paper, based on the context of the “COVID-19 pandemic”, combines the practical experience accumulated by the team in researching innovation alliances and solicits the opinions of industry experts. Referring to the parameter-setting principles of previous studies, the relevant parameters were set. Compared with academic and research institutions, the proportion of investment by enterprises in technology R&D is more prominent. Referring to the investment costs of the two entities in the alliance as noted by Hua and Wang (33), set c_b2_ = 30 and c_b3_ = 20. The value of c_b1_, as the total cost of the government's application of supply, demand, and environmental policies when actively participating in alliances, that is *k*_1_+*k*2+*p*+*rW*_2_+*rW*_3_. Opportunity loss is an important factor that urges multiple entities to participate in the alliance. Referring to the proportion of opportunity cost set by Xu et al. (34) and the emergency R&D budget from 2020 to 2025 in the report of the Ministry of Finance of China, *B*_1_ = 10, *B*_2_ = 8, and *B*_3_ = 6 are set. The active participation of all entities in the alliance will lead to high returns in terms of income. Referring to the setting of the income ratio of different entities and the government purchase mechanism in the study by Shen et al. (35), and combining with the actual data of the emergency R&D budget of the Ministry of Finance of China from 2020 to 2025, set *W*_1_ = 70, *W*_2_ = 60, *W*_3_ = 40, and *p* = 18. Referring to Chen's setting of government subsidies (36), set *k*_1_ = 24 and *k*_2_ = 18. Referring to the setting of the revenue coefficient by Wang et al. (37), the market revenue ratio λ of pharmaceutical enterprises in government purchases is set to be 0.7. Furthermore, since China does not have a unified tax subsidy policy, referring to the approximate median value of the current tax incentive coefficients for some enterprises, the tax incentive coefficient r is set as 0.1. The specific parameter settings are as follows: *c*_b2_ = 30, *B*_1_ = 10, *B*_2_ = 8, *B*_3_ = 6, *c*_b3_ = 20, *W*_1_ = 70, *W*_2_ = 60, *W*_3_ = 40, *p* = 18, *r* = 0.1, λ=0.7, *k*_1_ = 24, and *k*_2_ = 18.

### 5.1 Benchmark evolution path simulation analysis

**Figure 2:**
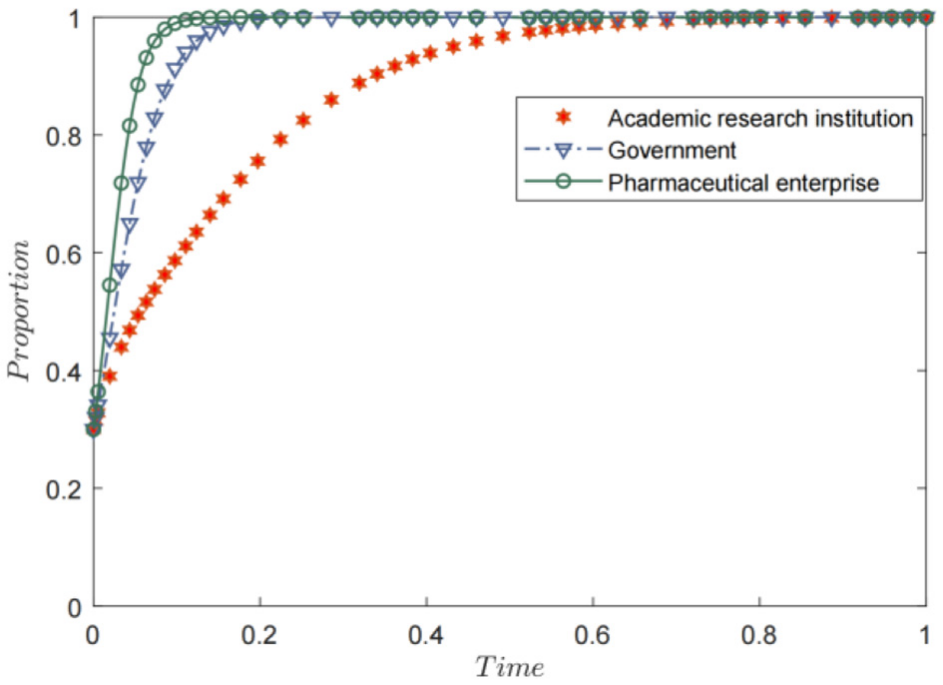
Benchmark evolution path simulation analysis.

Based on the stability analysis of the aforementioned equilibrium point, the ideal evolutionary stable equilibrium should be one where all the entities actively participate, that is, H8 (1, 1, 1), which condition *k*_1_+*k*_2_−*B*_1_+*p*+*rW*_2_+*rW*_3_<*W*_1_ is met, the system evolution path is shown in Figure 2. The final strategies of the government, pharmaceutical enterprises, and academic and research institutions converge to (1,1,1); that is, the government chooses active incentives, pharmaceutical enterprises choose active participation, and academic and research institutions choose active R&D. This result also verifies the derivation result of situation 3. It can be observed from Figure 2 that pharmaceutical enterprises tend to participate actively at the fastest speed, indicating that these enterprises have the best feedback effect on the market. Next is the government, indicating that it is an important driver of the public health technology R&D alliance. Following this, academic and research institutions indicate that policy-driven approaches are an important direction for optimizing their participation.

### 5.2 Simulation analysis of the influence of key factors

#### 5.2.1 The influence of government financial support on the evolution of technology R&D alliance relationship

**Figure 3:**
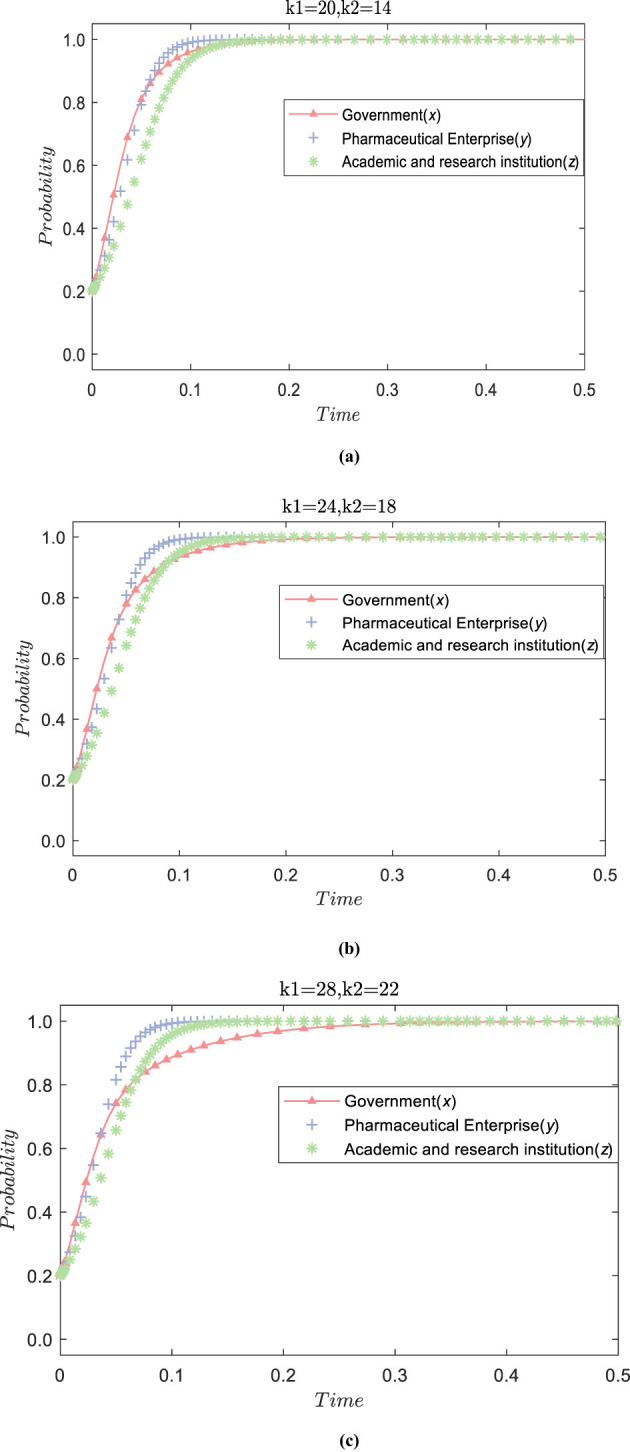
The impact of government funding incentives on the evolution of the tripartite subject strategy.

With other parameters remaining unchanged, the government's financial incentives for pharmaceutical enterprises and academic and research institutions are adjusted. The values are (*k*_1_ = 20, *k*_2_ = 14), (*k*_1_ = 24, *k*_2_ = 18), (*k*_1_ = 28, *k*_2_ =22). The simulation results are presented in Figure 3, whick **(a)** represents the value (*k*_1_ = 20, *k*_2_ = 14), **(b)** represents the value (*k*_1_ = 24, *k*_2_ = 18), and **(c)** represents the value (*k*_1_ = 28, *k*_2_ = 22). It can be seen that the government's stability strategy will eventually tend to adopt positive financial support. However, as the value of financial incentives increases, the government's convergence speed to positive strategy will slow down. This is because of the public nature and importance of public health technology R&D; the government will actively promote the construction of technology R&D alliances through financial support. Still, as financial incentives increase, it places certain pressure on government finances, resulting in a relatively slow pace of actively promoting the technology R&D alliance. Second, with the increase of government incentives, pharmaceutical enterprises and academic and research institutions choose to participate actively in the technology R&D alliance more quickly, gradually evolving into positive strategies. This indicates that under the government's financial incentives, the initiative of enterprises and academic and research institutions to participate in the technology R&D alliance is fully mobilized. The higher the financial incentive, the faster the two join the technology R&D alliance. Finally, under the influence of government funding, the strategies of the game subjects tend to favor active participation strategies. However, because pharmaceutical enterprises are market-oriented subjects, they are more sensitive to the incentive effect of funds than academic and research institutions and tend to adopt active strategies the fastest. This shows that when the government guides pharmaceutical enterprises and academic and research institutions to actively cooperate in public health science and technology research, it is necessary to control the range of financial incentives within a reasonable limit and adopt differentiated incentives according to the feedback effects of pharmaceutical enterprises and academic and research institutions.

#### 5.2.2 The influence of government purchase on the evolution of technology R&D alliance relationship

**Figure 4:**
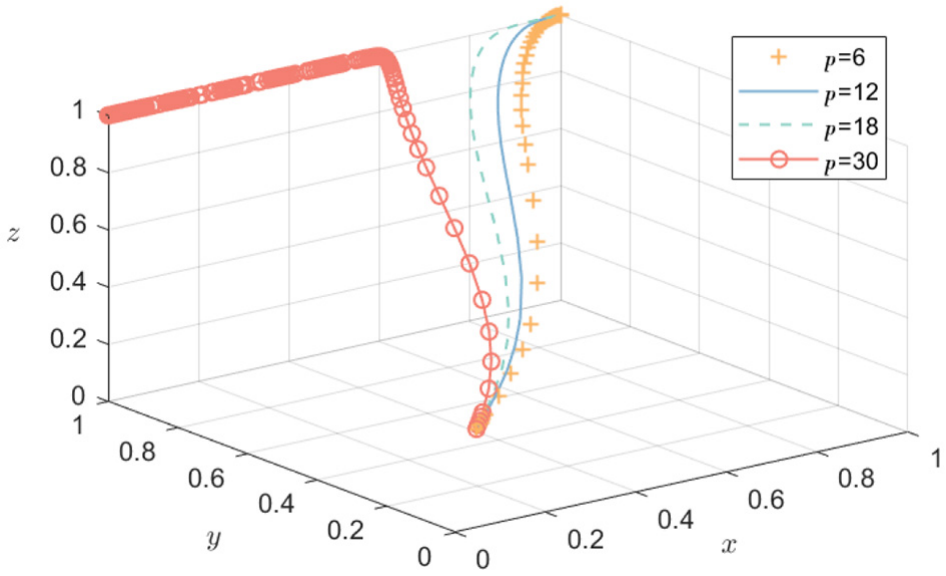
The influence of government purchase on the strategy of the three parties.

To observe the simulation of the impact of the government's purchase of public health products p on the participation of government, pharmaceutical enterprises, and academic and research institutions in emergency science and technology research, while ensuring that other parameters remain unchanged, the values assigned are *p* = 6, *p* = 12, p = 18, and *p* = 30. The evolution process of the strategies of the three parties is shown in Figure 4. It can be seen that there are differences in the evolution of the tripartite subject's strategies with respect to the government's purchase intensity. When p is 6, 12, and 18, the convergence of *x, y*, and *z* tends to 1. When p is 30, *x* converges to 0, while *y* and *z* converge to 1. As government purchases increase, the speed of convergence of pharmaceutical enterprises and academic research's willingness to actively participate in emergency science and technology research accelerates. However, when the government purchase intensity exceeds the critical point (such as *p* = 30), the government's own strategic choice changes. Due to the pressure of cost burden, it is no longer inclined to adopt a positive incentive strategy. Therefore, the government's purchase policy should remain within a reasonable boundary to stimulate pharmaceutical enterprises and academic and research institutions to actively participate in the scientific and technological research alliance, without becoming an economic burden that affects the government's own operation.

#### 5.2.3 The influence of government tax incentive coefficient on the evolution of technology R&D alliance relationship

**Figure 5:**
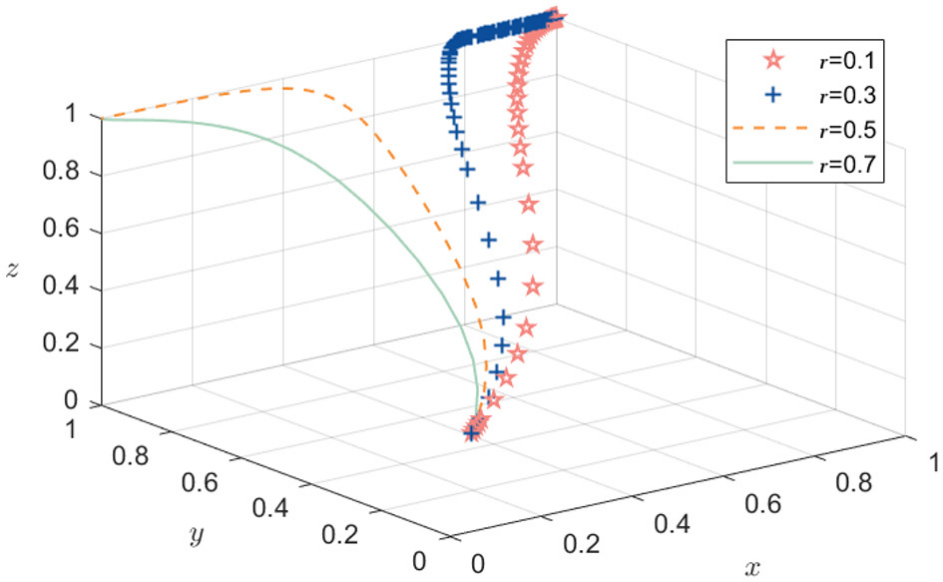
The impact of tax incentives on the three-party subject strategy.

Under the condition that other parameters remain unchanged, this paper analyzes the influence of changes in government tax incentives on the strategies of pharmaceutical enterprises and academic and research institutions, assigning them values of *r* = 0.1, *r* = 0.3, *r* = 0.5, and *r* = 0.7, respectively. The evolution path of the three-party game subjects is shown in Figure 5. It can be observed that when the tax incentive intensity is 0.1 and 0.3, the three-party strategies gradually evolve toward cooperation, ultimately stabilizing the three-party subject in a positive state. However, when the tax incentives are 0.5 and 0.7, although the stabilization strategy of enterprises and research institutions still tends to cooperate, the government's stabilization strategy shifts toward passive participation. The simulation results indicate that while the intensity of government tax incentives encourages multiple entities to engage in public health technology R&D alliances, excessive tax incentives can become a burden for the government's participation in these alliances, leading to a shift in the government's strategy from positive to negative. Therefore, in the management mechanism of the public alliance for public health science and technology, it is essential to leverage the government's tax incentives while also controlling the intensity to avoid ‘too much' effects of excessive government involvement.

#### 5.2.4 The impact of opportunity cost loss on the evolution of different agents ‘ strategies

**Figure 6:**
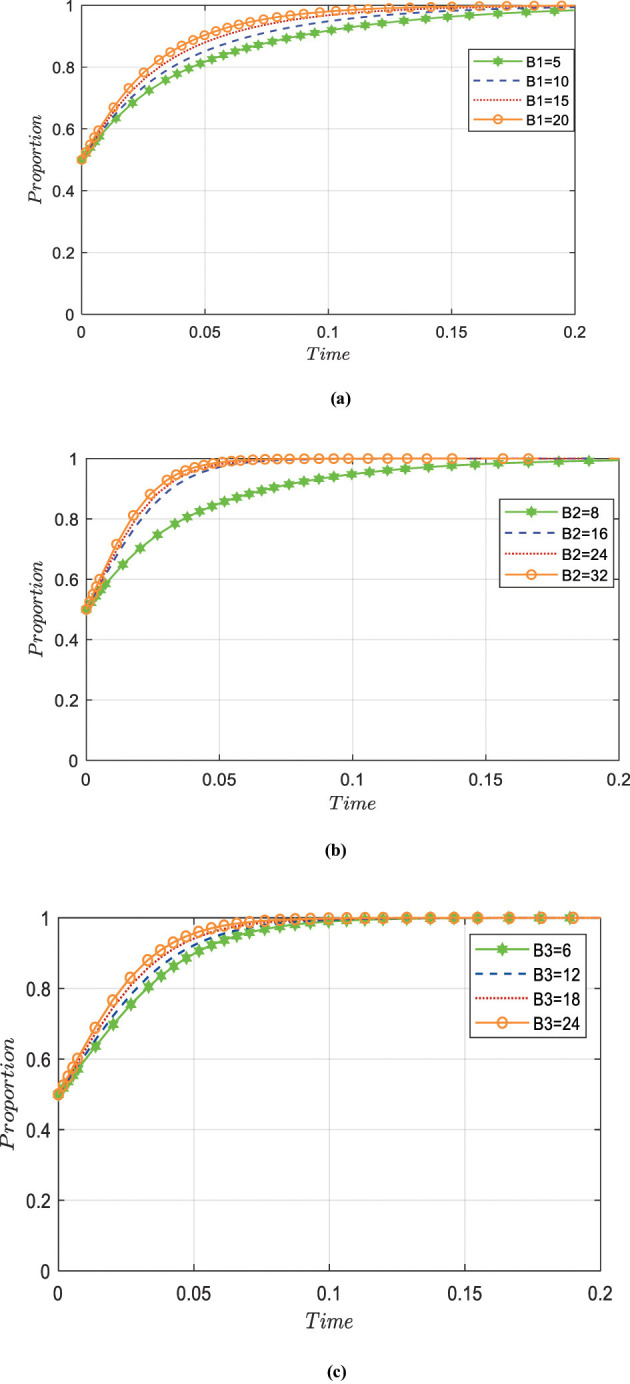
**(a)** The impact of opportunity cost changes on government strategies. **(b)** The impact of opportunity cost changes on pharmaceutical enterprise strategies. **(c)** The impact of opportunity cost changes on the strategies of academic and research institutions.

Figure 6a presents a simulation diagram illustrating the impact of opportunity cost loss B such as reputation, on government strategy selection when other parameters remain unchanged and public health subjects are not actively involved. It can be observed that as the value of *B*_1_ increases, the government's willingness to actively participate strengthens, indicating that internal factors such as the loss of government credibility and rising prevention and control costs will drive the government to adopt a series of policy tool combinations to promote the development of technology R&D alliances. Figures 6b, c shows the evolution results obtained by adjusting the values of B2 and B3 while keeping other parameters constant. It can be noted that as the value increases, the curve becomes steeper, and the speed of convergence to 1 accelerates; that is, the greater the loss of opportunity cost, the more pharmaceutical enterprises and research institutions will actively engage in emergency science and technology research strategies. Additionally, it should be noted that there are differences in the sensitivity of the three agents to the cost of opportunity loss. Among them, the evolution trajectory of pharmaceutical enterprises under different opportunity loss costs shows greater variation, indicating that pharmaceutical enterprises are more sensitive to the cost of opportunity loss.

## 6 Research conclusion and policy implication

### 6.1 Research conclusion

The public health technology R&D alliance is focused on sudden public health events. Its key characteristics of short research and development time and high trial and error costs impose greater requirements for the effectiveness of government policy tool combinations. Therefore, from the perspective of government policy tool integration, and based on evolutionary game theory, this paper considers the government, pharmaceutical enterprises, and academic and research institutions as the main players in the game. It constructs an evolutionary game model of the public health technology R&D alliance, analyzes the evolutionary game under different policy incentives, and verifies the findings through numerical simulation. The results show that first, there is a balanced and stable strategy that aligns with the interests of the government, pharmaceutical enterprises, and academic and research institutions. The combination of effective policy tools can promote the establishment of a stable state of emergency science and technology research that is actively supported by the government and enthusiastically participated in by pharmaceutical enterprises and academic and research institutions. Second, different policy tools have varying stimulating effects on the game players. The demand-side product purchase policy can enhance the willingness of multiple players to engage in the public health technology R&D alliance, followed by supply-side financial incentives and environmental-side tax incentives, with enterprises being more sensitive to the feedback from policy tools. Third, the strength of policy tools must be controlled within the scope of science. While the public health technology R&D alliance should leverage the core guidance of the government, it should also activate the contributions of social entities such as pharmaceutical enterprises and academic and research institutions. On the one hand, it should maximize the collaborative efforts of multiple stakeholders, and on the other hand, it should avoid the ‘too little' effect caused by overburdening the government.

### 6.2 Policy implication

Establish a symbiotic and coordinated technology R&D alliance. The formation of a technology R&D alliance is a crucial means to effectively address sudden public health events. It requires the government to actively encourage collaboration, engage in top-level design, and integrate public health resources to build an emergency science and technology research platform. Additionally, developing a normalized public health technology R&D system is essential for gradually improving the efficiency and systematization of emergency science and technology research. Policy tools such as financial incentives, product purchases, and tax incentives should be used to guide emergency management departments, leading pharmaceutical enterprises, key research laboratories, and other related entities to form a public health technology R&D alliance. In the event of a sudden public health crisis, this alliance can quickly mobilize to conduct emergency science and technology research and achieve a rapid response mechanism.

Improve the scientific and efficient interest coordination mechanism. In the face of sudden public health incidents, exploring the common interests of the government, pharmaceutical enterprises, and academic and research institutions is important for the rapid response to emergency science and technology. First, the government should establish reasonable intervals and values according to the specific conditions of each public health issue, combined with quantitative simulation tools. It should adopt appropriate incentive models and safeguard policies for public health entities to improve their initiative to participate in emergency science and technology research. Second, to address the difficulties and interest demands encountered during public health technology R&D, as well as the additional costs associated with major science and technology projects, a corresponding interest compensation mechanism should be implemented to improve the efficiency of technological innovation and the transformation of achievements among public health entities. In addition, further consideration should be given to the impact of opportunity costs on the strategic choices of stakeholders. It is necessary to adjust subsidy measures appropriately to promote the enthusiasm and sense of responsibility of pharmaceutical enterprises and academic and research institutions in drug research and development and key technology research through reputation incentives.

Leverage the combined synergy of policy tools. Due to the differences in the characteristics of multi-alliance subjects and the effects of different policy tools, the combined effects of these tools should be explored in practice. First, the government should exercise reasonable control over the implementation of policy tools, enhance the guiding and stimulating functions of social capital, and avoid the cost burden and crowding-out effect caused by excessive participation. Second, for pharmaceutical enterprises and research institutions, the initial focus should be on strengthening the stimulating effects of policy tools on economic outcomes, while later efforts should shift toward utilizing tax incentives to promote the formation of a positive ecosystem in which multiple stakeholders actively engage in the public health technology R&D alliance.

## Data Availability

The original contributions presented in the study are included in the article/supplementary material, further inquiries can be directed to the corresponding author.
